# Innovative Endoscopic Management of Recurrent Gastrogastric Fistula Post-Roux-en-Y Gastric Bypass Patient Using Cardiac Septal Occluder and Lumen-Apposing Metal Stent

**DOI:** 10.14309/crj.0000000000001642

**Published:** 2025-03-17

**Authors:** Ajay Gade, Kalpit Devani

**Affiliations:** 1 Department of Gastroenterology, Prisma Health, University of South Carolina, Greenville, SC

**Keywords:** gastrogastric (GG) fistula, Roux-en-Y gastric bypass (RYGB)' endoscopic intervention, recurrent GG fistula, gastrojejunal (GJ) stricture, cardiac septal occlude Amplatzer, septal dccluder Axios stent, lumen-apposing metal stent (LAMS)

## Abstract

Gastrogastric fistula (GGF) is a rare complication of post-Roux-en-Y gastric bypass, often challenging to manage. This case report presents a 63-year-old patient with recurrent GGF and gastrojejunal stricture, refractory to prior endoscopic treatments. Using a minimally invasive approach with atrial septal defect closure device and a lumen-apposing metal stent, the fistula was successfully closed, and stricture relieved. The patient reported complete symptom resolution at follow-up. This innovative approach of combining a cardiac septal occluder and lumen-apposing metal stent for management of complex GGF cases offers a promising alternative to surgical interventions.

## INTRODUCTION

Gastrogastric fistula (GGF) is a rare but significant late complication following Roux-en-Y gastric bypass (RYGB)^1^ associated with abdominal pain, acid reflux, weight regain, and diabetes recurrence. Up to 6% of post-RYGB patients develop GGF requiring endoscopic or surgical intervention.^2^ Endoscopic interventions, though less invasive, can have recurrence especially with concomitant gastrojejunal (GJ) strictures that exacerbate symptoms and complicate management.^1^ This report details the novel use of a cardiac septal occluder (CSO) and lumen-apposing metal stent (LAMS) to manage a recurrent GGF and GJ stricture, offering a minimally invasive solution with promising outcomes.

## CASE REPORT

A 63-year-old woman with a history of RYGB performed for class-III obesity presented with recurrent GGF for 4 years. The GGF was identified 5 years after the RYGB surgery. Despite multiple endoscopic treatments, including argon plasma coagulation (APC) and over-the-scope clip placement at an outside facility, her symptoms of abdominal pain, nausea, vomiting, and weight regain persisted.

Upon referral to our facility, she underwent through-the-scope suturing with an X-tack device in a figure-of-8 pattern, achieving temporary symptom relief. However, symptoms recurred within 6 weeks. Follow-up endoscopy revealed GGF recurrence, and she underwent APC ablation (80 W, 1 L/min) and full-thickness suturing with an Apollo endoscopic suturing device. Fistula was successfully closed, and subsequent upper gastrointestinal series confirmed the closure. However, the patient's symptoms recurred, and imaging reconfirmed the fistula.

Repeat evaluation revealed a 14-mm GGF and GJ outlet stricture near the fistulous tract, with residual suture material from prior closures. Initial endoscopies identified a GJ stricture, but prior attempts did not include stricture dilations which led to persistent symptoms and recurrent fistula. We opted for endoscopic closure using an atrial septal defect (ASD) closure device and concomitant GJ stricture management to offload pressure on the fistula for durable closure.

### Step-by-step procedure

**Figure 1.**
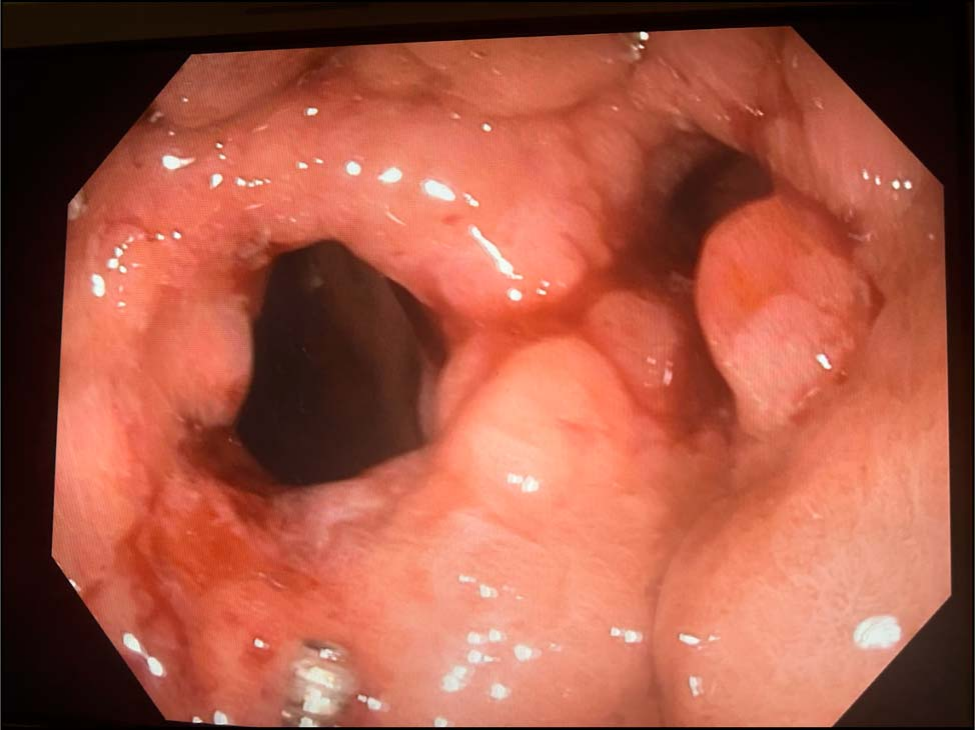
Showing a fistulous tract between gastric pouch and excluded stomach on the left side. Right side opening is the strictured opening of Gastrojejunostomy.

**Figure 2.**
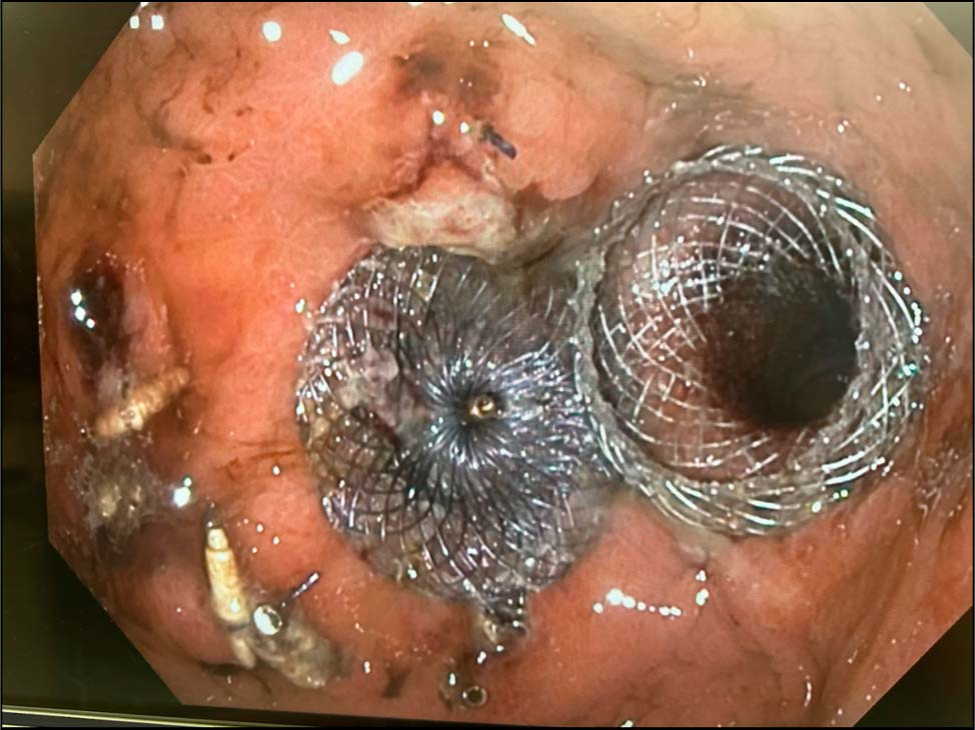
Showing devices cardiac septal occluder used in closing the fistula (left side) and lumenapposing metal stent (right side) across gastrojejunal anastomosis outlet.

1. Fistula Ablation: APC was applied to the fistula at 80 W with 1 L/min flow to resurface mucosa.
2. ASD Closure Device Placement: A 0.035-inch guidewire was introduced into the Roux limb to aid LAMS placement. Considering the fistula size (14 mm), a 16-mm ASD closure device was chosen to avoid occluding the GJ outlet. The device was back-loaded into a double-channel therapeutic scope. Using a rat-tooth grasper, the device was successfully deployed, sealing the fistula with its dual-disc design (Figure 1).
3. LAMS Placement: A 15 mm × 10 mm LAMS was deployed over the guidewire, with the proximal flange in the pouch and the distal flange in the Roux limb, ensuring GJ patency (Figure 2).

The patient was placed on a liquid diet for 1 week and prescribed omeprazole 40 mg twice daily for 4 weeks. At a 4-week televisit follow-up, she reported complete symptom resolution including no further nausea, vomiting, abdominal pain, or weight gain, with stable glycemic control. She relocated before follow-up endoscopy, leaving LAMS removal pending.

## DISCUSSION

The combination of a CSO and LAMS reflects an innovative, minimally invasive approach to managing recurrent GGFs and GJ strictures. GJ stricture management is essential in these cases to reduce pressure on the GGF, a key factor in preventing recurrence.

GGF, an abnormal connection between stomach segments post-RYGB, can lead to weight regain, ulceration, and infection. Its closure is critical.^1^ The CSO, typically used for ASDs or patent foramen ovale, has been repurposed for gastrointestinal applications fistulas.^1^ Its dual-disc structure creates a secure seal, promoting healing and permanent closure.^3^

LAMS, a specialized endoscopic stent, has broad applications, including pseudocyst drainage, bypassing gastric outlet obstructions, and managing leaks or strictures.^3^ Its large lumen ensures effective drainage, while wide flanges prevent migration. For GJ stricture cases, LAMS establishes a durable pathway, reducing fistula pressure, and recurrence risk.^4–6^

This case highlights the successful combination of an Amplatzer Septal Occluder for durable GGF closure and LAMS for maintaining GJ patency. The dual-disc occluder's adaptability ensured effective sealing, while the LAMS addressed stricture-related complications, achieving symptom resolution in a single session.

Prior studies report that the success rate for endoscopic closure of GGF with CSO ranges from 70% to 85%, with complications including device migration and occlusion. LAMS placement for GJ stricture has demonstrated a success rate of approximately 75%–90%, with potential complications such as bleeding, stent migration, and restenosis.^7–10^ The estimated cost of a CSO ranges from $3,000 to $6,000,^11^ while a LAMS device can cost up to $20,000.^12^ While these costs may seem significant, they must be weighed against the potential expense of surgical intervention and hospitalization for recurrent complications.

Combining a CSO with LAMS offers a minimally invasive, effective strategy for recurrent GGFs and GJ strictures post-RYGB. This dual-device approach provided symptom relief, durable fistula closure, and GJ patency. Future studies should explore long-term outcomes to establish this as a viable alternative to surgery for complex GGFs.

## DISCLOSURES

Author contributions: A. Gade primarily contributed to conceptualizing the case report, gathering clinical data, conducting the literature review, and drafting the case report and is the article guarantor. K. Devani: Senior author who provided oversight and guidance throughout the preparation of the case report, reviewed the manuscript critically, and ensured its clinical and academic integrity.

Financial disclosure: None to report.

Informed consent was obtained for this case report.
